# Predicting molecular subtype in breast cancer using deep learning on mammography images

**DOI:** 10.3389/fonc.2025.1638212

**Published:** 2025-09-16

**Authors:** Yunzhao Luo, Jing Wei, Yang Gu, Chuang Zhu, Feng Xu

**Affiliations:** 1 Department of Breast Surgery, Beijing Chao-Yang Hospital, Capital Medical University, Beijing, China; 2 School of Artificial Intelligence, Beijing University of Posts and Telecommunications, Beijing, China

**Keywords:** breast cancer, molecular subtypes, mammography, deep learning, DenseNet121-CBAM

## Abstract

**Objectives:**

This study aimed to develop and evaluate a deep learning model for predicting molecular subtypes of breast cancer using conventional mammography images, offering a potential alternative to invasive diagnostic techniques.

**Methods:**

A retrospective analysis was conducted on 390 patients with pathologically confirmed invasive breast cancer who underwent preoperative mammography. The proposed DenseNet121-CBAM model, integrating Convolutional Block Attention Modules (CBAM) with DenseNet121, was trained and validated for binary (Luminal vs. non-Luminal, HER2-positive vs. HER2-negative, triple-negative vs. non-TN) and multiclass (Luminal A, Luminal B, HER2+/HR+, HER2+/HR−, TN) classification tasks. Performance metrics included AUC, accuracy, sensitivity, specificity, and interpretability via Grad-CAM heatmaps.

**Results:**

The model achieved AUCs of 0.759 (Luminal vs. non-Luminal), 0.658 (HER2 status), and 0.668 (TN vs. non-TN) in the independent test set. For multiclass classification, the AUC was 0.649, with superior performance in distinguishing HER2+/HR− (AUC = 0.78) and triple-negative (AUC = 0.72) subtypes. Attention heatmaps highlighted peritumoral regions as critical discriminative features.

**Conclusion:**

The DenseNet121-CBAM model demonstrates promising capability in predicting breast cancer molecular subtypes from mammography, offering a non-invasive alternative to biopsy.

## 1 Introduction

Despite the decline of death rate in recent years through earlier detection and advancement in treatment, breast cancer remains ranking second in cancer-related mortality among women worldwide and the leading cause of cancer death in Black and Hispanic women (1). In order to achieve personalized precision medicine, it is essential to crystallize molecular subtype before starting treatment, guiding physicians to tailor treatments and inform patients of their prognosis. Since the latest St. Gallen International Consensus Conference (2023), the molecular classification of invasive breast carcinoma has been divided into four main subtypes based on the expression of immunohistochemical markers, including estrogen receptor (ER), progesterone receptor (PR), human epidermal growth factor receptor 2 (HER2) and Ki-67, which is an indicator of cell proliferation (2). Different molecular subtypes lead to disparate prognosis, and the recommended treatment strategy for each molecular subtype is also different. For example, patients with Luminal A breast cancer often have a good prognosis, and postoperative endocrine therapy alone is sufficient to inhibit tumor recurrence and metastasis for part of them. Whereas Triple Negative breast cancers are very aggressive, chemotherapy and novel targeted drugs are the only means for both neoadjuvant therapy and adjuvant therapy. Besides, HER2 positive breast cancers have their specific targeted therapies which have been updated over years (3).

However, the process of detecting the molecular subtypes remains quite expensive and time-consuming at present. Firstly, a tumor biopsy is needed, which may cause several possible complications, such as hemorrhage, infection (4) or even needle tract metastases with low possibility (5). In order to further describe tumor characterization, multigenetic assay represented by Oncotype DX, microRNA sequencing and proteomics are also necessary (6). Even so, error on the technique procedure and the heterogeneity of the tumor are inevitable, more tools are needed to comprehensively and efficiently excavate the molecular feature of breast cancer.

Certain mutations, as well as the status of ER, PR, and HER2, are widely utilized in diagnostic practices across many countries. These parameters hold significant prognostic value in the disease and demonstrate correlations with mammographic findings. As a widespread and non-invasive examination method, mammography can display the overall characteristic information of the tumor, and describe the tumor microenvironment which can’t be fully provided by needle biopsy. A significant correlation was observed between the enzymatic activities of LDH and CAT in tumor tissue and mammographic characteristics (7). Patients exhibiting high mammographic density demonstrated significantly elevated pSTAT3 tumor expression levels compared to those with low mammographic density (8). Among hormone receptor-negative/HER2+ patients, mammography demonstrated the highest prevalence of calcifications, predominantly manifesting as pleomorphic or branching calcifications (9). Moreover, previous studies had demonstrated that tumor shape, microcalcifications and margin characteristics are strongly correlated with molecular subtype (10, 11), suggesting the possibility of predicting breast cancer molecular subtype by mammogram.

With advances in computer algorithms, major breakthroughs have been made in the recognition and analysis of medical images by artificial intelligence, turning the above assumption into reality. Zhu et al. (12) distinguished two classifications of immunohistochemical results using contrast-enhanced mammography (CEM) based on selected radiomics features and support vector machine classifier. The binary classification capability of the model in the external test was satisfactory with AUC between 0.69 to 0.83. Deng et al. (13) aimed to predict HER2-positive status by manually extracting quantitative radiomics features and using Gradient Boosting Machine as classifier, which achieved an AUC of 0.702 in the external validation cohort. Considering the limitation of the radiomics approaches, which limits tumor analysis to handcrafted features, few studies were conducted using deep learning methods. Qian et al. (14) used an end-to-end learning convolutional neural network to do biomarker status prediction on CEM. However, there was no external verification, and the deep learning model performed best in HER2 status prediction with an AUC of 0.67 while the accuracy dropping to 60%.

There are three main limitations to the mentioned studies: firstly, CEM equipment is relatively high-end which has not been popularized in primary hospitals. Secondly, manually feature extraction probably miss important information in the mammogram, more deep information can be extracted by neural networks. Thirdly, the absence of interpretability failed to visualize the deeper features extracted by the model. In this paper, we will take above limitations into account, building a deep learning model to predict different molecular subtypes of breast cancer based on conventional mammography images. Our study includes testing classification strategies (both binary and multi-category) and model interpretation.

## 2 Patients and methods

### 2.1 Patients

The institutional review board of Beijing Chaoyang Hospital affiliated to Capital Medical University granted approval to this study (approval code: 2022-4-19-1). We retrospectively analyzed data from pathologically confirmed breast cancer (BC) patients who received preoperative mammography from January 2018 to December 2023. The molecular subtyping into five categories was determined according to the St. Gallen International Consensus Conference 2023 criteria, based on immunohistochemical analysis of postoperative pathological specimens.

The detailed inclusion criteria were as follows: 1) they were diagnosed with primary invasive breast cancer by histological examination, 2) their molecular subtypes were determined by postoperative histopathological examination (gold standard), and 3) their preoperative mammographic imaging (CC or MLO views) exhibited a macroscopically detectable tumor mass. The exclusion criteria included the following: 1) with inflammatory breast cancer, bilateral or pathologically heterogeneous lesions, 2) history of radiotherapy, chemotherapy or anti-HER2 therapy, 3) their clinical information was incomplete, 4) they underwent invasive procedures including biopsy or surgical resection within a week before mammography. A total of 390 BC patients with mammographically visible lesions were enrolled for analysis. The patient recruitment workflow is shown in **Supplementary Figure 1**. Complete clinical characteristics of all enrolled patients are provided in **Supplementary Data 1**.

### 2.2 Data preprocessing

The mammographic images were acquired using the Hologic full-field digital mammography system with a spatial resolution of 7 lp/mm. All images were stored in DICOM format. In order to reduce heterogeneity between observers, two qualified radiologists independently examined and annotated all identifiable tumor areas in each CC or MLO images (by ITK-SNAP Software) while remaining unaware of patient information. We extracted the annotated tumor regions as the regions of interest (ROIs). Considering the potential influence of peri-tumoral background signals on molecular subtype prediction, we expanded each ROI outward by a specified pixel value (bound size) and subsequently adjusted them to square dimensions(224×224 pixels). Examples of the original and scaled images are shown in **Supplementary Figure 2**. We employed simple random oversampling to address class imbalance in the dataset. The study collected a total of 762 eligible mammography images from 390 patients. For the Luminal binary classification (Luminal: 501 images; non-Luminal: 261 images), the oversampling rate was set at 1.3. The TNBC binary classification (TNBC: 104 images; non-TNBC: 658 images) used an oversampling rate of 1.7. For the HER2 binary classification (HER2 positive: 157 images; HER2 negative: 605 images), the oversampling rate was established at 1.5. In the five-class classification (Luminal A: 200 images, Luminal B: 301 images, HER2+/HR+: 107 images, HER2+/HR-: 50 images, TNBC: 104 images), we applied an oversampling rate of 1.5. Data augmentation through geometric transformations was further employed to increase sample diversity and improve model generalizability, including: random horizontal flipping (with a 50% probability), vertical flipping (also with a 50% probability), random rotation within a range of ±20°, as well as random horizontal shearing transformations with an angular range of ±10°.

### 2.3 Deep learning model development

Our proposed DenseNet-CBAM deep learning architecture was designed to classify mammography images into breast cancer molecular subtypes. The study comprised three binary classification tasks (Luminal vs non-Luminal, triple-negative (TN) vs non-TN, HER2-positive vs HER2-negative) and one multiclass classification task (Luminal A, Luminal B, HER2-positive/hormone receptor-positive (HER2+/HR+), HER2-positive/hormone receptor-negative (HER2+/HR−), TNBC).

#### 2.3.1 Feature extraction

Through comparative experiments with various convolutional neural network (CNN) architectures—including but not limited to Simple CNN, ResNet101, DenseNet121, MobileNetV2, MOB-CBAM (MobileNet-V3 integrated with CBAM), and ViT-B/16—DenseNet121 was ultimately selected as the backbone network for our proposed algorithm. Since mainstream CNN models are typically pretrained on three-channel (RGB) input images whereas mammography images are single-channel grayscale, our algorithm proposed a channel-adaptive pretrained weight allocation strategy to better leverage pretrained weights for classification guidance. The channel-adaptive pretrained weight allocation strategy adapts ImageNet (3-channel) pretrained weights to single-channel medical images by averaging the weights across the three input channels.

#### 2.3.2 CBAM

Inspired by MOB-CBAM’s implementation of channel attention mechanisms, we enhanced DenseNet121 by integrating Convolutional Block Attention Modules (CBAM) after its first three dense blocks. CBAM, originally proposed by Woo et al. (15), sequentially applies channel and spatial attention weights to amplify critical features while suppressing less informative regions. In Channel Attention Module (CAM), the input first undergoes global average pooling to generate a channel descriptor vector. This vector is then transformed by a multilayer perceptron (MLP) to produce channel attention weights, which are normalized to the range [0, 1] via a sigmoid function. Notably, in the DenseNet121-CBAM architecture, the MLP is implemented using two 1×1 convolutional layers, compressing the spatial dimensions of each channel to 1×1, with ReLU as the activation function. In Spatial Attention Module (SAM), the channel attention weights are multiplied with the original input, and the result is processed through a convolutional layer to generate spatial attention weights. These weights are then normalized to [0, 1] using a sigmoid function. In this study, a convolutional layer with a kernel size of 7 is specifically employed in SAM. This architectural modification yields our proposed DenseNet121-CBAM model, which demonstrates improved feature representation capacity and generalization performance.

#### 2.3.3 Model training and testing

**Figure 1:**
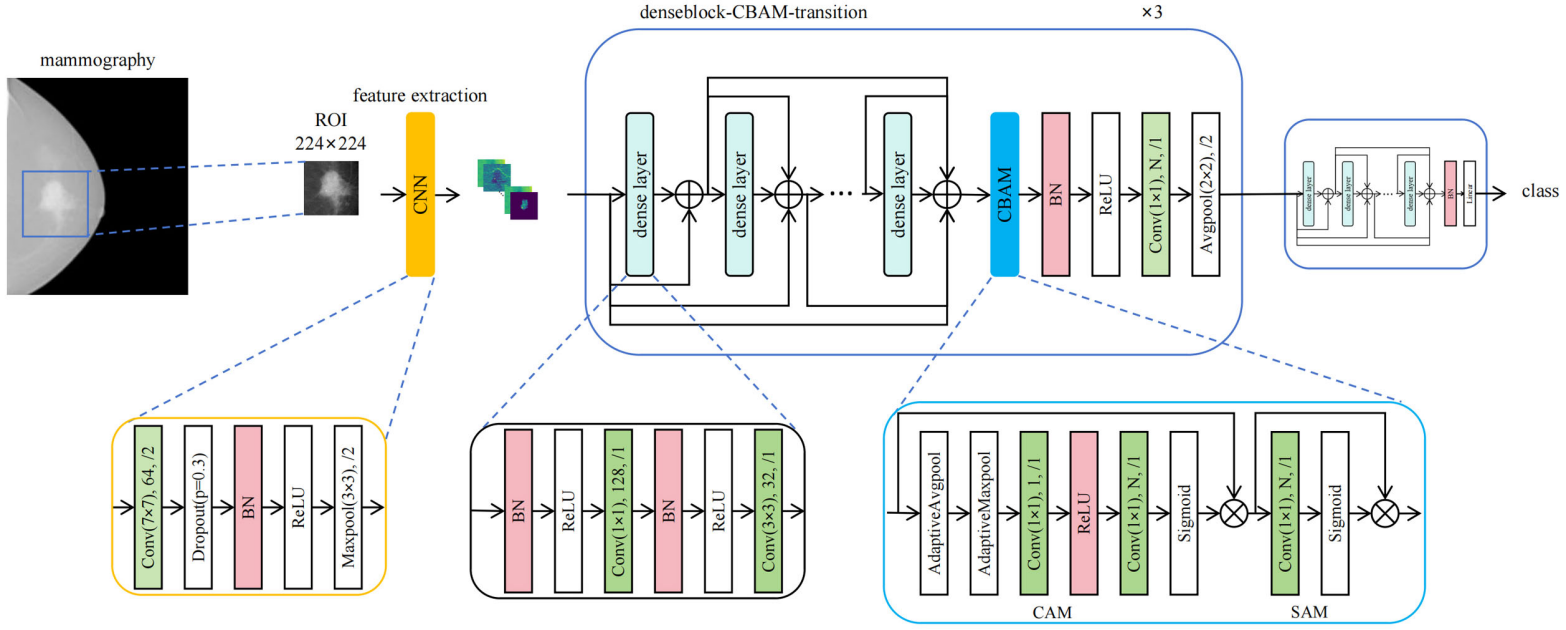
Architecture of the DenseNet121-CBAM model for predicting molecular subtypes from mammography images.

We randomly divided the mammogram ROI patches into training cohort and independent test cohort with the ratio of 4:1. A 5-fold cross-validation scheme was implemented on the training cohort, wherein the training cohort were randomly partitioned into 5 mutually exclusive subsets (folds). In each iteration, 4 folds were combined for training while the remaining fold served as validation. Model performance was ultimately assessed by aggregating results from all 5 validation cycles. We used the fold with the highest AUC to represent the model’s optimal performance. During model training, we observed that the performance peaked at a bound size of 100. Therefore, we set the bound size to 100 in our DenseNet121-CBAM model. We employed AdamW (16) as the optimizer and implemented Cosine Annealing schedule (17) for learning rate adaptation. The detailed model configuration and hyperparameters are as follows: Initial learning rate: 0.0001; Loss function: Cross-entropy loss; Batch size: 8; Momentum: 0.9; Weight decay: 0.005; Dropout rate: 0.3; Number of training epochs: 300. The structure of the proposed DenseNet121-CBAM model is shown in **Figure 1**.

### 2.4 Interpretability of deep learning model

We visualized the important regions that were more associated with molecular subtypes. For input images, we generated and visualized activation maps from each convolutional layer to elucidate how the model progressively extracts hierarchical features (such as edges, textures and shapes). Utilizing Gradient-weighted Class Activation Mapping (Grad-CAM) (18), we localized class-discriminative regions by computing gradient-weighted spatial importance. The Grad-CAM visualization was implemented using the pytorch_grad_cam library with five key components: (1) Dynamic target layer selection via recursive traversal of model architecture using dot-notation; (2) Standardized image preprocessing including resizing (224×224), ImageNet normalization, and single-to-three channel conversion; (3) Heatmap generation through gradient-weighted class activation mapping from specified convolutional layers; (4) Visualization by superimposing grayscale heatmaps on original images to highlight decision-relevant regions; (5) Systematic output generation saving high-resolution comparative visualizations with prediction metadata. The resulting grayscale heatmap was superimposed onto the original image, producing an attention map that visually highlights the model’s focal regions during classification decisions.

We have publicly released the full source code (https://github.com/LemonWei111/molsub), including detailed documentation (README) for replication.

Trained model weights can be seen in: https://drive.google.com/drive/folders/1rYldK579H_BmYjJNUrBdBWUenpg89E_k?usp=sharing.

Anonymized original data examples (https://drive.google.com/drive/folders/1aVJjBz9f3nkS-HtQ3xevpfWhtnHUafi2?usp=sharing) and full preprocessed datasets (https://drive.google.com/drive/folders/1E_zJ66rPS6bFNrO_sTY7tFTXe6WZIEkn?usp=sharing) were provided.

### 2.5 Statistical analysis

The SPSS software was utilized for statistical analysis in the study. For normally distributed continuous variables, one-way ANOVA (for normally distributed data with equal variance, e.g. Age) or Kruskal-Wallis tests (when these assumptions were violated, e.g. Number of lymph node metastasis) were performed to compare means between different molecular subtype groups. Categorical variables (e.g. Clinical stages, Nerve invasion, Vascular invasion, Ki67) were analyzed using Pearson’s chi-square tests when all expected cell frequencies were≥5; otherwise, likelihood ratio chi-square tests were employed (e.g. T grade, N grade). The implementation utilized PyCharm (Python 3.10) as the integrated development environment and PyTorch 2.5.1 as the deep learning framework. All computational tasks were accelerated using an NVIDIA GeForce RTX 3090 GPU deployed on a remote server. To evaluate the performance of DenseNet121-CBAM, we employed accuracy (ACC) and the area under the receiver operating characteristic curve (AUC-ROC) as primary metrics. DeLong’s test and McNemar’s test were used to compare model performance (AUCs and ACC) across tasks and architectures. The other measurements like sensitivity (SENS), specificity (SPEC), positive predictive value (PPV), and negative predictive value (NPV) were also used to estimate the model performance. *P*-value <0.05 was considered statistically significant.

## 3 Results

### 3.1 Clinical characteristics

A total of 390 BC patients with mammographically visible lesions were enrolled for analysis, stratified by molecular subtype as follows: Luminal A (n=102), Luminal B (n=155), HER2-positive/HR-positive (n=54), HER2-positive/HR-negative (n=27), and TN (n=52). Among the comparisons of the five molecular subtypes, age (*F* = 4.639, Partial *η*^2^ = 0.046, *p* = 0.001), number of lymph node metastases (*H* = 12.147, Partial *η*^2^ = 0.021, *p* = 0.016), vascular invasion status (*χ*2 = 11.968, Cramer’s V = 0.175, p=0.018), and Ki-67 expression (*χ*2 = 369.032, Cramer’s V = 0.688, p<0.001) showed statistically significant differences. After Bonferroni correction for p-values, a significant difference was only observed between the age of Luminal A and HER2+/HR+ subtypes (adjusted p < 0.05). As for number of lymph node metastases and vascular invasion, none of the pairwise comparisons between molecular subtypes showed significant differences after Bonferroni correction (all adjusted p > 0.05). Tumor T grade, N grade, clinical stages, and presence of neural invasion were not associated with molecular subtypes. Detailed clinical characteristics of the patients are presented in **Table 1**. All patients were randomly divided into training set (n=312) and an independent test set (n=78) at a 4:1 ratio. Five-fold cross-validation was applied to the training set, reserving one fold (20%) for validation.

**Table 1:** Patients clinical characteristics.

Clinical features	LuminalA	LuminalB	HER2+(HR+)	HER2+(HR-)	Triple-negative breast cancer (TN)	Statistical value and effect size	*p*
	(n=102)	(n=155)	(n=54)	(n=27)	(n=52)		
Age (mean ± SD)	65.12 ± 12.38	61.52 ± 11.44	56.85 ± 12.56	63.33 ± 10.56	60.42 ± 12.09	F=4.639 Partial *η*^2^ = 0.046	**0.001**
T grade (%)						χ2 = 9.390 df=12 Cramer’s V = 0.09	0.669
1	53 (52.0)	63 (40.6)	19 (35.2)	11 (40.7)	24 (46.2)		
2	45 (44.1)	82 (52.9)	32 (59.3)	15 (55.6)	26 (50.0)		
3	3 (2.9)	10 (6.5)	3 (5.6)	1 (3.7)	2 (3.8)		
4	1 (1.0)	0 (0.0)	0 (0.0)	0 (0.0)	0 (0.0)		
N grade (%)						χ2 = 14.545 df=12 Cramer’s V = 0.11	0.267
0	63 (61.8)	75 (48.4)	27 (50.0)	17 (63.0)	35 (67.3)		
1	24 (23.5)	45 (29.0)	12 (22.2)	5 (18.5)	11 (21.2)		
2	11 (10.8)	19 (12.3)	10 (18.5)	2 (7.4)	4 (7.7)		
3	4 (3.9)	16 (10.3)	5 (9.3)	3 (11.1)	2 (3.8)		
Clinical stages (%)						χ2 = 12.980 df=8 Cramer’s V = 0.129	0.113
1	39 (38.2)	36 (23.2)	12 (22.2)	10 (37.0)	18 (34.6)		
2	46 (45.1)	81 (52.3)	27 (50.0)	12 (44.4)	28 (53.8)		
3	17 (16.7)	38 (24.5)	15 (27.8)	5 (18.5)	6 (11.5)		
Number of lymph node metastasis Median M (P25, P75)	0.000(0.0,2.0)	1.000(0.0,3.0)	0.500(0.0,5.3)	0.000(0.0,2.0)	0.000(0.0,1.0)	H=12.147 Partial *η*^2^ = 0.021	**0.016**
Nerve invasion (%)						χ2 = 9.083 df=4 Cramer’s V = 0.153	0.059
No	80 (78.4)	120 (77.4)	48 (88.9)	20 (74.1)	48 (92.3)		
Yes	22 (21.6)	35 (22.6)	6 (11.1)	7 (25.9)	4 (7.7)		
Vascular invasion (%)						χ2 = 11.968 df=4 Cramer’s V = 0.175	0.018
No	80 (78.4)	96 (61.9)	33 (61.1)	21 (77.8)	40 (76.9)		
Yes	22 (21.6)	59 (38.1)	21 (38.9)	6 (22.2)	12 (23.1)		
Ki67 (%)						χ2 = 369.032 df=8 Cramer’s V = 0.688	<0.001
≤15%	100 (98.0)	0 (0.0)	7 (13.0)	1 (3.7)	5 (9.6)		
15-30%	2 (2.0)	104 (67.1)	21 (38.9)	11 (40.7)	10 (19.2)		
>30%	0 (0.0)	51 (32.9)	26 (48.1)	15 (55.6)	37 (71.2)		

Bold values indicate a significant overall difference across the five groups (p < 0.05).

### 3.2 Convolutional neural network model selection

Evaluating the three binary classification tasks and one five-class classification task collectively, DenseNet121-CBAM demonstrated superior performance over both MOB-CBAM and Simple CNN in both validation set and independent test set, as detailed in **Supplementary Figure 3**. Furthermore, considering other DenseNet121-based structure, we compared DenseNet121-CBAM with DenseNet121-pre, DenseNet121-tcAta, and DenseNet121. The results demonstrated that DenseNet121-CBAM achieved optimal performance among the compared models, with the most stable AUC outputs across different dataset partitions, and either DeLong’s test (for AUC comparison) or McNemar’s test (for accuracy comparison) yielding *P*-value <0.05 in all pairwise comparisons. Therefore, we selected DenseNet121-CBAM as the final model for our experiment. Detailed results are provided in **Supplementary Figure 4**.

### 3.3 Prediction value of DenseNet121-CBAM between different molecular subtypes

**Figure 2:**
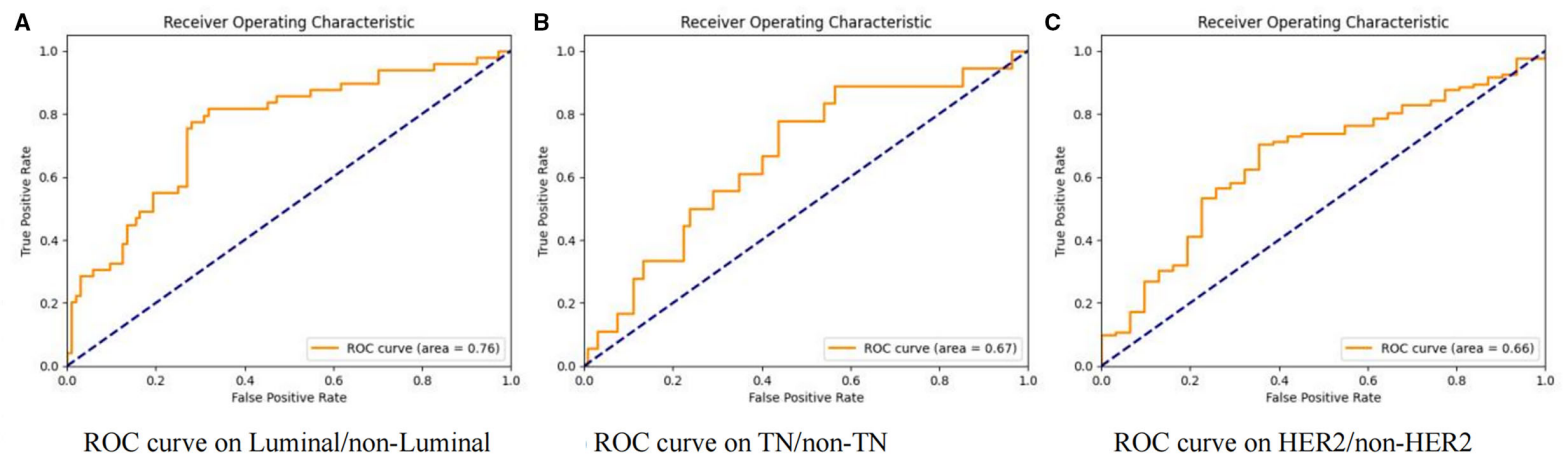
Receiver operating characteristic (ROC) curves of the DenseNet121-CBAM model for molecular subtype discrimination. Three binary classification tasks: **(A)** Luminal vs. non-Luminal, **(B)** TN vs. non-TN, and **(C)** HER2 vs. non-HER2.

Our study initially performed model training and validation on three binary classification tasks: Luminal vs non-Luminal subtypes, HER2-positive vs HER2-negative status, and TN versus non-TN breast cancer. Results from the independent test cohort demonstrated that DenseNet121-CBAM achieved optimal performance in distinguishing Luminal from non-Luminal subtypes, with an optimal AUC of 0.7592 (Mean AUC ± standard deviation over 5-fold cross-validation: 0.6979 ± 0.0416). The corresponding ROC curve is presented in **Figure 2A**. Furthermore, the model exhibited specificity (SPEC) and negative predictive value (NPV) exceeding 70%, along with accuracy (ACC) and sensitivity (SENS) surpassing 60%, confirming its robust discriminatory capability for Luminal-type breast cancer classification. The detailed statistical results are summarized in **Table 2**.

**Table 2:** Model performance for Luminal subtype classification.

Evaluation index	Validation set	Test set
AUC	0.7666	0.7592
ACC (%)	0.626 (0.534, 0.717)	0.669 (0.635, 0.704)
SENS (%)	0.508 (0.347, 0.669)	0.604 (0.380, 0.828)
SPEC (%)	0.697 (0.531, 0.862)	0.700 (0.595, 0.805)
PPV (%)	0.486 (0.313, 0.658)	0.486 (0.448, 0.524)
NPV (%)	0.727 (0.649, 0.805)	0.798 (0.721, 0.874)

95% confidence intervals are included in brackets.

**Figure 3:**
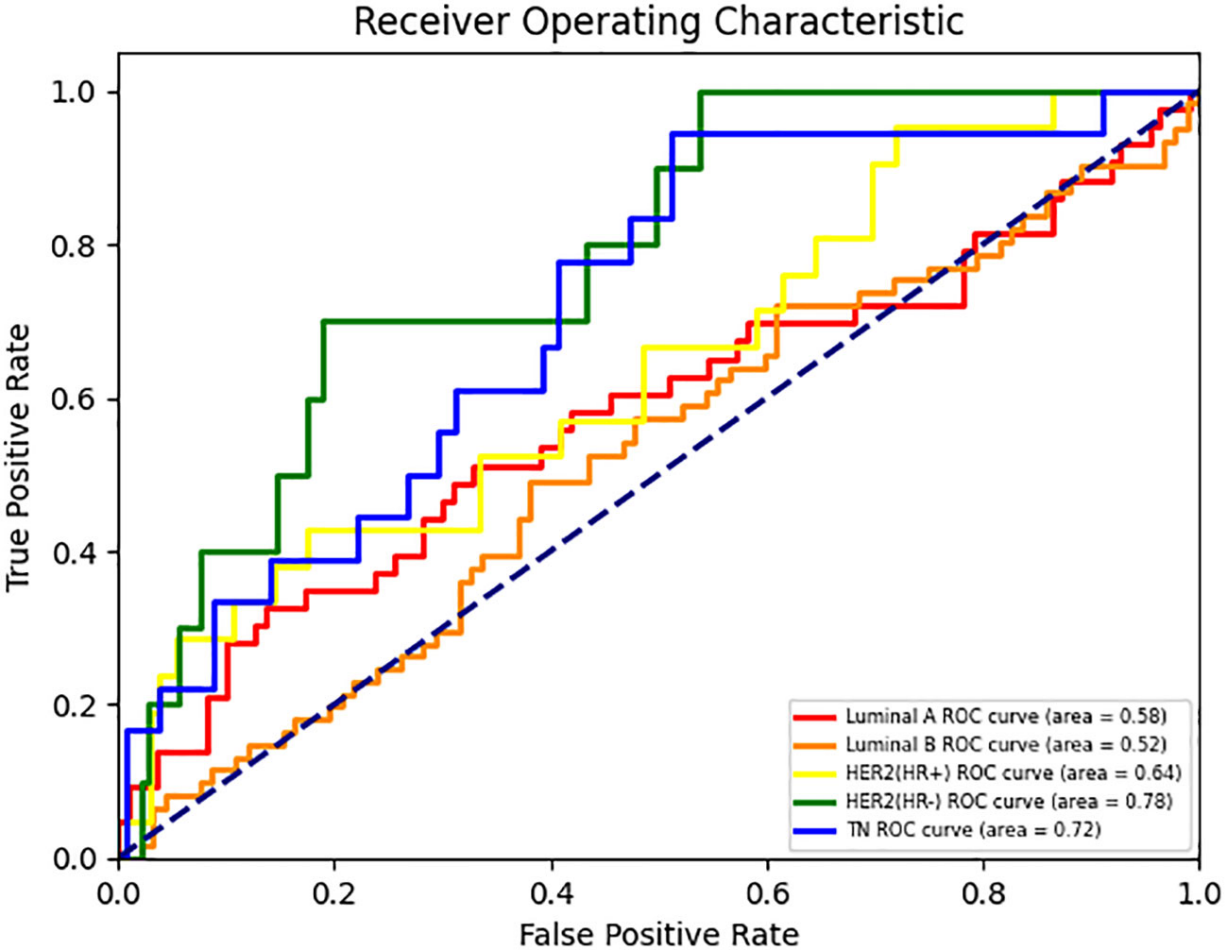
ROC curves for each class in the DenseNet121-CBAM model’s five-class molecular subtype classification task.

In the two binary classification tasks of TN vs non-TN and HER2-positive vs HER2-negative breast cancer, the model achieved optimal AUC values exceeding 0.65 in the independent test cohort (Mean AUC ± standard deviation over 5-fold cross-validation: 0.6209 ± 0.0588 and 0.6344 ± 0.0383), with corresponding ACC of 0.769 and 0.697, respectively. The corresponding ROC curves are presented in **Figures 2B, C**. Furthermore, we evaluated the performance of the DenseNet121-CBAM model in a five-class classification task encompassing the following molecular subtypes of breast cancer: Luminal A, Luminal B, HER2+/HR+, HER2+/HR− and TN. The detailed statistical results are summarized in **Table 3**. The results demonstrated that the model attained an optimal AUC of 0.6494 in the independent test set (Mean AUC ± standard deviation over 5-fold cross-validation: 0.6219 ± 0.0236), indicating moderate discriminative capability among these subtypes. The ROC curves for each molecular subtype are collectively presented in **Figure 3**. The model demonstrated relatively poor discrimination for Luminal subtypes, while showing better performance in distinguishing HER2+/HR− and TN subtypes, with AUC values of 0.78 and 0.72, respectively.

**Table 3:** Model performance in classifying TN (binary), HER2 status (binary), and five molecular subtypes (5-category).

Evaluation index	TN		HER2		Five molecular subtypes	
	V-set	T-set	V-set	T-set	V-set	T-set
AUC	0.7164	0.6679	0.7402	0.6584	0.6542	0.6494
ACC (%)	0.754 (0.704, 0.803)	0.769 (0.697, 0.841)	0.708 (0.644, 0.771)	0.697 (0.629, 0.764)	0.287 (0.217, 0.358)	0.325 (0.294, 0.357)
SENS (%)	0.364 (0.208, 0.521)	0.300 (0.157, 0.443)	0.800 (0.671, 0.928)	0.797 (0.688, 0.905)	0.429 (0.213, 0.646)	0.455 (0.289, 0.621)
SPEC (%)	0.819 (0.758, 0.879)	0.831 (0.741, 0.922)	0.390 (0.240, 0.540)	0.303 (0.169, 0.438)	0.607 (0.446, 0.768)	0.584 (0.499, 0.669)
PPV (%)	0.249 (0.174, 0.323)	0.198 (0.137, 0.260)	0.834 (0.772, 0.895)	0.818 (0.799, 0.838)	0.548 (0.376, 0.719)	0.599 (0.545, 0.653)
NPV (%)	0.887 (0.852, 0.921)	0.899 (0.883, 0.915)	0.362 (0.187, 0.538)	0.281 (0.229, 0.333)	0.489 (0.393, 0.584)	0.439 (0.378, 0.501)

95% confidence intervals are included in brackets.

TN, Triple-negative breast cancer; V-set, Validation set; T-set, Test set.

### 3.4 Interpretability of DenseNet121-CBAM model

**Figure 4:**
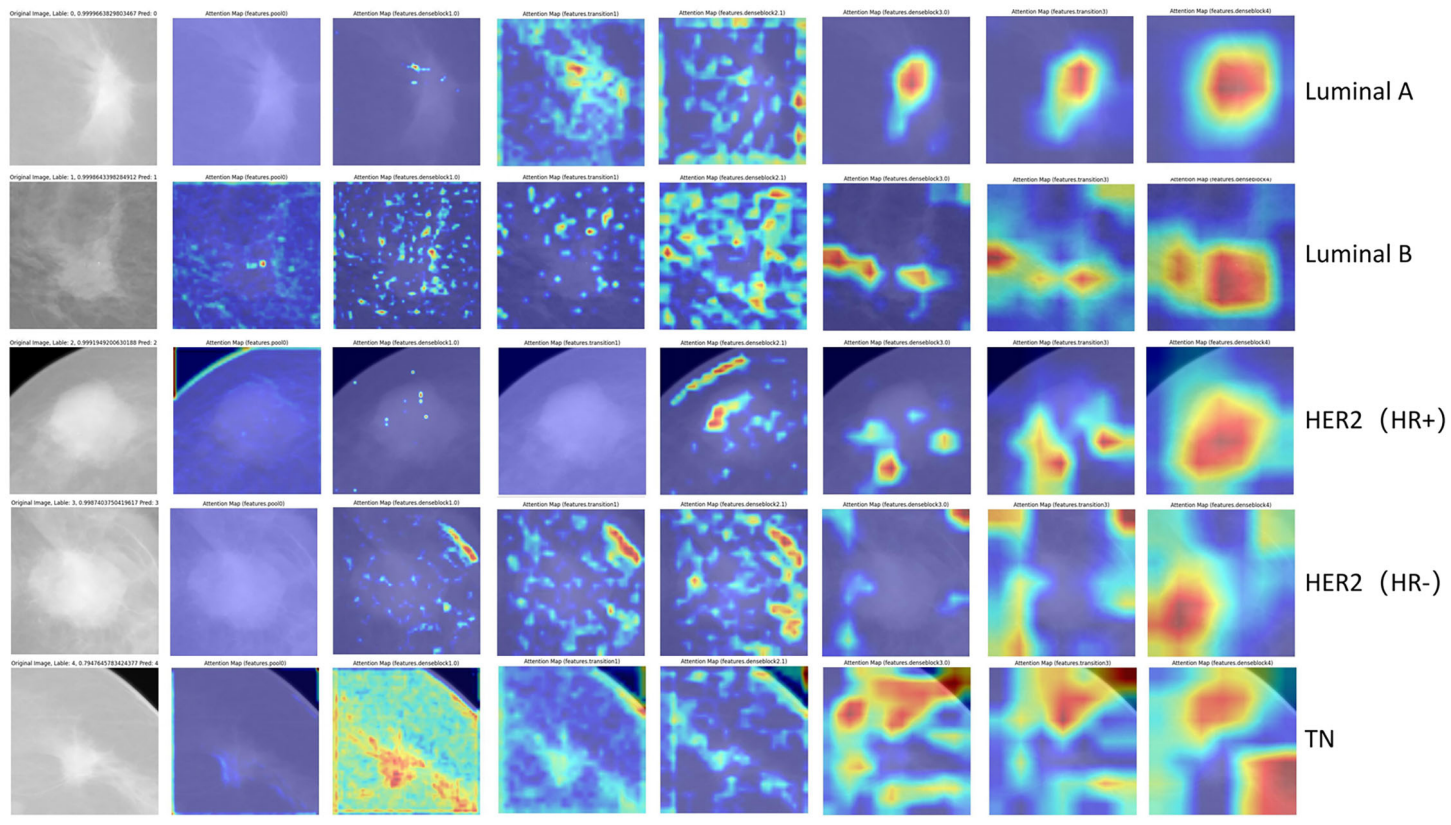
Visualization of representative images with correct classification across different molecular subtypes. The red regions show greater contribution to the final classification.

We employ visual attention heatmaps to highlight the most salient regions in each convolutional layer, demonstrating how our DL model progressively focuses on the tumor from the original input image. As shown in **Figure 4**, the heatmap identifies critical regions with red patches, while blue areas indicate non-salient regions. Notably, in the five-category classification task, the model demonstrated superior discriminative performance for TNBC and HER2+/HR− subtypes. Attention heatmap analysis of select TNBC and HER2+/HR−mammograms revealed predominant activation at the tumor periphery (peritumoral stroma), suggesting the potential existence of subvisual tumor-associated characteristics, including peritumoral immune microenvironment alterations. This observation merits further histopathological validation.

Comparative visualization for each binary classification task is provided in **Supplementary Figure 5**. As shown in **Supplementary Figures 5A, B**, the model’s attention for TN subtypes appears more dispersed compared to non-TN cases, which aligns with clinical observations of TN tumors—typically exhibiting irregular morphology, crab-like infiltration, and spiculated margins. This suggests that the key discriminative features for TN classification may reside primarily in the tumor periphery rather than the core region. **Supplementary Figures 5C, D** shows Luminal cases exhibit centripetal attention patterns (model’s attention originates from edge to core), implying central tumor features may drive Luminal classification, unlike TN’s edge-dependent signatures. Discrepancies in model attention localization between CC and MLO projections for HER2+ cases (vs. TN’s consistent edge-predominant pattern) may indicate HER2 tumor heterogeneity (**Supplementary Figures 5E, F**).

## 4 Discussion

We investigated the application of deep learning for predicting breast cancer molecular subtypes directly from mammographic images. Our methodology employed a pre-trained DenseNet121-CBAM architecture and evaluated its performance through both binary and multiclass classification paradigms.

As early as 2019, Ma et al. employed radiomics approaches to perform binary classification of molecular subtypes using mammographic images. The researchers extracted 39 quantitative radiomic features from segmented lesion areas, achieving AUC values over 0.78 across three binary classification tasks with accuracy rates exceeding 0.74 (19). Subsequent studies have further validated that manually extracted radiomic features from mammograms can accurately predict TNBC subtypes, achieving an AUC of 0.84 (20). However, in 2024, Duan et al. also employed radiomics analysis of mammograms for ER status prediction, yet achieved substantially inferior performance (AUC/accuracy: 0.61/0.57) (21). The limitations of radiomics stem from its dependence on manual feature extraction, which introduces excessive subjectivity. Their reliance on expert-defined features means they may not represent the optimal feature quantification method for the imminent differentiated tasks (22).

Given these constraints, we elected to utilize CNNs as the backbone of our predictive model, thereby eliminating human-intervened feature selection. In the context of model architecture selection, we systematically evaluated various CNNs as feature extraction modules, among which DenseNet121 exhibited superior performance. The DenseNet architecture, initially developed by Huang Gao and colleagues in 2016, was specifically engineered to optimize feature reuse and propagation efficiency through its innovative connectivity pattern (23). In practical applications, Adedigba et al. achieved breast cancer diagnosis using deep learning models with a small dataset of mammographic images, where the DenseNet model demonstrated optimal performance with an accuracy of 0.998 (24). In 2024, Nissar et al. developed a lightweight dual-channel attention-based deep learning model named MOB-CBAM, which integrates MobileNet-V3 architecture with convolutional block attention modules (CBAM). Through comprehensive validation on the CMMD mammography dataset, the model demonstrated exceptional efficacy in classifying masses and calcifications in mammograms, achieving a remarkable accuracy rate of 98% (25). Building upon these previous experimental outcomes, we integrated DenseNet121 with CBAM, thereby proposing the DenseNet121-CBAM architecture. Our results demonstrated that this hybrid model exhibits superior performance relative to other DenseNet121-based structures.

In image preprocessing part, due to the varying sizes of the annotated regions across samples, different scaling ratios were applied during the resizing step to achieve a uniform input size (224×224). This variation in scaling may introduce geometric distortions—such as blurring from up-sampling or detail loss from down-sampling—which could potentially affect model predictions, especially for samples with extreme aspect ratios or small object sizes. To mitigate such effects, we employed standard normalization and comprehensive data augmentation (including random cropping, flipping, and color jittering), which help improve the model’s robustness to scale variations. Furthermore, the use of deep architectures with strong feature abstraction capabilities contributes to learning scale-invariant representations to some extent. Moreover, our clinical data indicate no statistically significant differences in tumor size across molecular subtypes (**Table 1**; T grade: χ²=9.390, p=0.669), suggesting that tumor size (and thus scaling ratio) is unlikely to significantly impact molecular subtype prediction. We acknowledge that scale-aware or adaptive resizing strategies (e.g., multi-scale training or adaptive pooling) could be explored in future work to further reduce bias introduced by non-uniform scaling.

Among binary classification tasks, the differentiation between Luminal and non-Luminal subtypes demonstrated superior performance (AUC = 0.759), suggesting distinct imaging characteristics detectable by our deep learning model, while HER2-positive versus HER2-negative classification yielded the lowest discriminative capacity (AUC = 0.658). However, our results diverge from those reported by Mota et al. in the OPTIMAM mammography public database, where their ResNet-101 architecture achieved optimal discriminative performance for HER2 status (AUC = 0.733) but limited efficacy in Luminal subtype classification (AUC = 0.531) (26). The discrepancy may be attributed to either (1) inherent differences in data distribution between the public database and our institutional cohort, or (2) variations in ROI feature extraction capabilities across different convolutional neural network (CNN) architectures. Regarding the relatively poorer performance of the HER2 classification task, we attribute this to the following factors. Firstly, HER2-positive tumors can co-express HR (HER2+/HR+) or lack HR (HER2+/HR−), leading to tumor heterogeneity and divergent imaging phenotypes (27). For instance, HER2+/HR+ tumors often resemble Luminal subtypes in mammographic features, while HER2+/HR− tumors may display aggressive features like pleomorphic calcifications or irregular margins (9). This heterogeneity could dilute the model’s ability to generalize HER2-specific features. Secondly, HER2-positive cases constituted only 20.8% (81/390) of our dataset, with HER2+/HR− being particularly rare (6.9%, 27/390). This relatively limited sample size may explain the observed performance differences, while deep learning typically requires large datasets for stable training, radiomics can construct effective models with smaller samples (28). This sample size constraint likely accounts for the superior performance of radiomics in previous studies (19, 20). Thirdly, Zhu et al. (12) demonstrated superior HER2 prediction (AUC = 0.83) using CEM, suggesting that iodine-based contrast enhancement may better capture HER2-related angiogenic features. The superior performance of CEM may be attributed to its ability to provide more distinct imaging features associated with HER2 status (29). Our use of conventional mammography (without contrast) likely contributed to the performance gap.

In the domain of five-category molecular subtype prediction, only two research teams to date have conducted multiclass classification tasks on public mammography datasets using deep learning models. Mota et al., as noted above, utilized the OPTIMAM database to classify tumors into five subtypes (Luminal A, Luminal B1, Luminal B2, HER2-enriched, TNBC), achieved an average AUC of 0.606 (26). Ben Rabah et al. utilized the Chinese Mammography Database (CMMD) for five-category breast lesion classification (benign, Luminal A, Luminal B, HER2+, TNBC). Model performance demonstrated substantial dependence on clinical data integration, with AUC dropping from 0.88 (mammography combined with clinical data) to 0.61 (mammography only) (30). Our model achieved an AUC of 0.65 for five-category molecular subtypes classification, demonstrating superior performance compared to existing deep learning models trained on public mammography databases. However, the ACC remained suboptimal in both validation and test cohorts, which we attribute to class imbalance in our dataset—a common challenge in clinically collected samples. Specifically, the overrepresentation of Luminal A/B (26.2%/39.7%) versus the underrepresentation of HER2+/HR− (6.9%) likely contributed to this performance discrepancy.

For model visualization, we illustrate the progressive localization of tumor-associated discriminative regions across consecutive convolutional layers. Notably, in the five-class classification task, the attention heatmaps of TNBC and HER2+/HR− subtypes—exhibiting superior interclass discriminability—predominantly highlighted peritumoral stromal regions rather than the tumor parenchyma itself. Regarding the heterogeneity in discriminative region distribution, prior studies have demonstrated that different deep learning architectures exhibit distinct attention patterns in mammographic classification tasks. For instance, baseline CNNs and AGN4V predominantly focus on local features (e.g., lesion regions), whereas Transformer-based MaMVT tend to prioritize global contextual features (e.g., entire breast tissue) (31). Within an identical deep learning architecture, the observed divergence in attention regions across molecular subtypes may be attributed to intrinsic tumor biology and tumor-immune microenvironment heterogeneity. Immunosuppressive cell populations (e.g., Tregs, MDSCs, Th2 cells, M2 macrophages) demonstrate higher infiltration in ER-negative and TN subtypes, whereas NK cells and cytotoxic T lymphocytes—cell types associated with antitumor activity—are more abundant in ER-positive breast carcinomas (32). The latest research showed that peritumor breast adipose-derived secretome from obesity patients is a strong inducer of TNBC cell invasiveness and JAG1 expression (33). Relevant studies have further revealed that TNBC promotes the transdifferentiation of adipocyte stem cells into myofibroblasts through zinc-α-2-glycoprotein secretion, suggesting the existence of a unique peritumoral adipose microenvironment in TNBC (34). Whether the observed differences in attention regions across molecular subtypes truly result from peritumoral cell distribution heterogeneity requires future validation through immunohistochemical analysis and peritumoral tissue single-cell RNA sequencing.

Our deep learning model enables molecular subtype prediction from mammographic images, which could be integrated into existing diagnostic workflows as a pre-biopsy classification tool. Patients will receive a molecular subtype prediction along with their mammography report, providing them with psychological preparedness for future diagnostics and treatment. Aggressive subtype predictions will prompt patient attention to necessary invasive biopsy procedures. Furthermore, the model’s classification output serves as an adjunctive tool for pathological assessment by pathologists. Notably, the model-identified regions of interest surrounding TNBC and HER2+/HR− tumors may serve as critical imaging biomarkers for assessing tumor aggressiveness. However, there are also some limitations in our study. First, the retrospective design lacks prospective and external validation cohorts to rigorously assess model generalizability. Second, we treated each patient’s CC and MLO views as independent inputs, missing opportunities to improve model performance through view-integrated prediction. Finally, the observed concentration of heatmap-activated regions in peritumoral areas warrants further mechanistic investigation. To address the limitations of retrospective design and strengthen generalizability, we propose a three-step validation strategy: (1) Collaborating with two additional medical centers to compile an independent external validation cohort, ensuring diversity in demographics and imaging protocols; (2) Designing a multicenter prospective trial to compare model predictions against postoperative pathology in real-time; and (3) Benchmarking performance on public datasets (OPTIMAM and CMMD) to assess cross-institutional robustness. These efforts will validate clinical applicability and will be completed in our future work. Moreover, mammography presents inherent limitations compared to MRI and ultrasound, particularly in the Chinese population where breasts typically exhibit lower fat content and higher glandular tissue density. This dense tissue composition reduces mammographic sensitivity, making ultrasound a more suitable primary imaging modality for many Chinese women. Relevant studies have confirmed that radiomics analysis of breast ultrasound images in Chinese women demonstrates predictive efficacy for ER, PR, HER2, and Ki-67 status, with AUC values all exceeding 0.7 (35). And the combination with contrast-enhanced ultrasound significantly improves the accuracy and AUC of radiomics-based prediction for molecular subtypes (36, 37). Regarding our model’s suboptimal HER2 status discrimination, multiparametric MRI may offer enhanced predictive value for HER2 expression assessment (38, 39). Future studies should investigate multimodal approaches combining mammographic patterns with ultrasound and multiparametric MRI features.

## 5 Conclusion

Our study developed a DenseNet121-CBAM model that demonstrates promising capability in predicting breast cancer molecular subtypes from mammography, providing a non-invasive alternative to biopsy and optimizing clinical workflows. In binary classification tasks, the model showed optimal performance in distinguishing Luminal subtypes, achieving an AUC of 0.7592 on the independent test set. For five-category classification, the model exhibited particularly strong predictive performance for HER2+/HR− and TNBC subtypes. Attention heatmaps revealed that the model’s discriminative regions were primarily located at tumor margins, suggesting HER2+/HR− and TNBC molecular subtypes may be associated with peritumoral cellular microenvironments.

## Data Availability

The original contributions presented in the study are included in the article/**Supplementary Material**. Further inquiries can be directed to the corresponding authors.
