# Supplementary figures and images for: Predicting molecular subtype in breast cancer using deep learning on mammography images
Source: Front Oncol. 2025 Sep 16;15:1638212. doi: 10.3389/fonc.2025.1638212 (PMC12479247; doi:10.3389/fonc.2025.1638212)

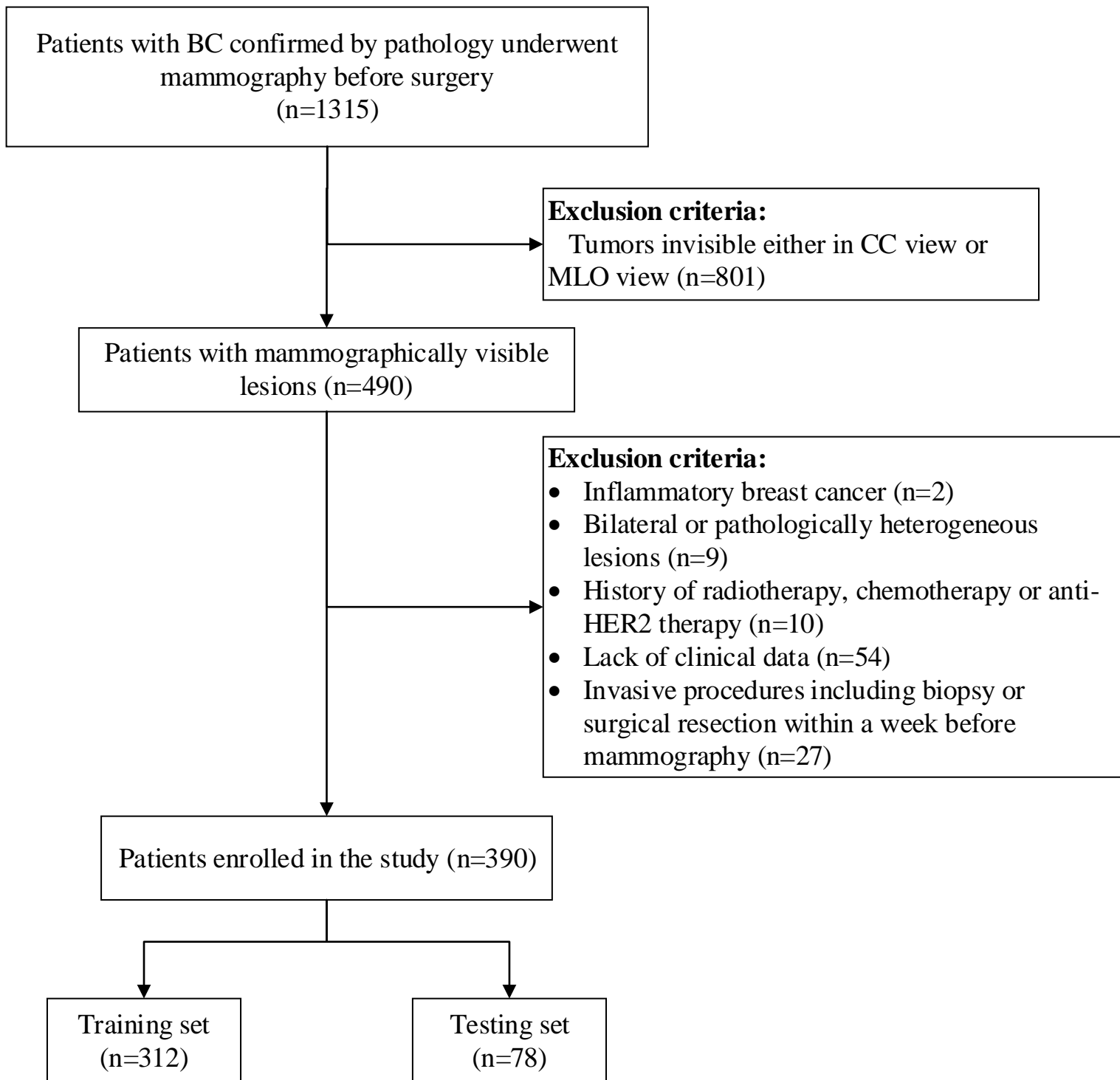

Supplement: Supplementary file 1 [file DataSheet1.pdf]

Supplementary Figure 2. Examples of the original and scaled images

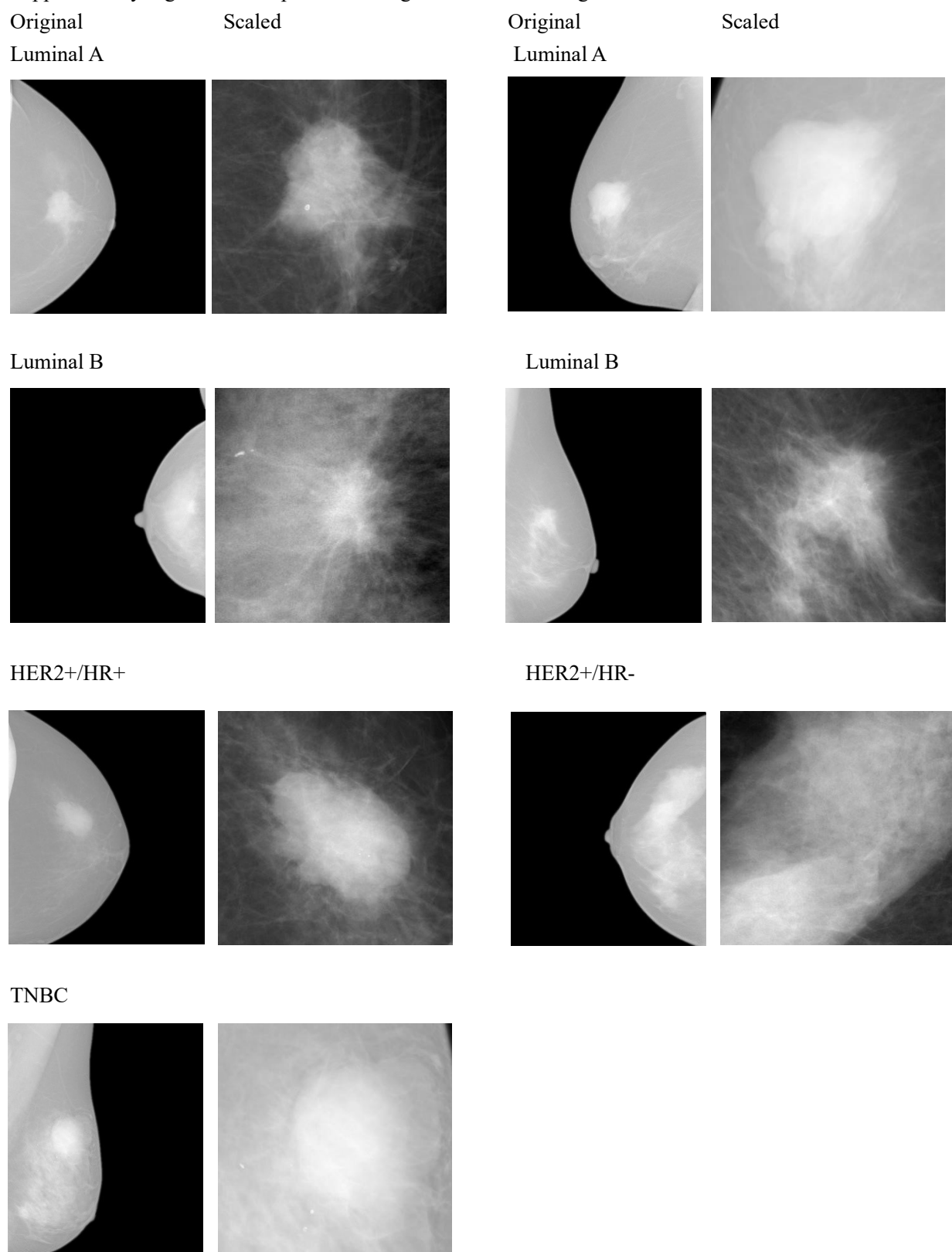

Supplement: Supplementary file 2 [file DataSheet2.pdf]

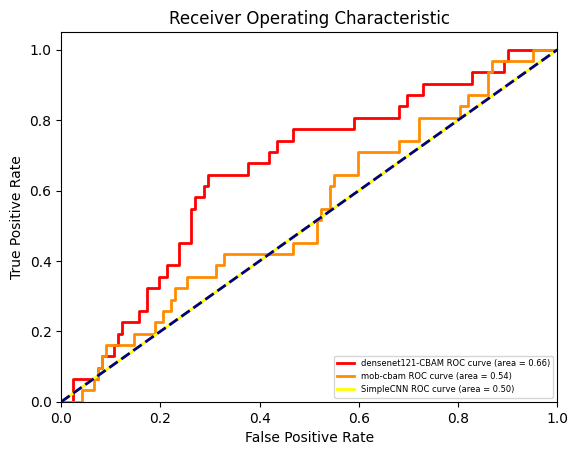

Supplement: Supplementary file 3 [file DataSheet3.zip › Sp1.densenet121-cbam、mob-cbam、cnn/binary-HER2.png]

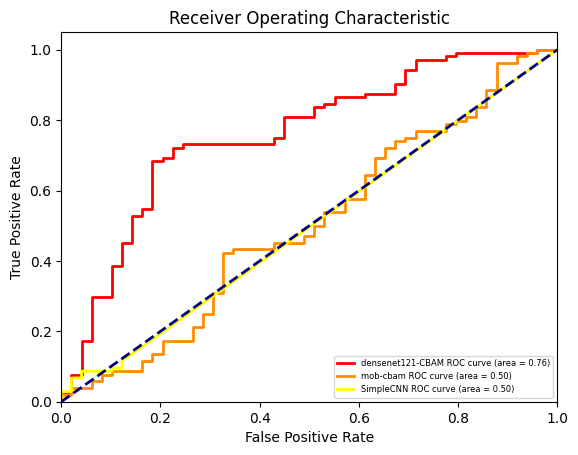

Supplement: Supplementary file 3 [file DataSheet3.zip › Sp1.densenet121-cbam、mob-cbam、cnn/binary-Luminal.png]

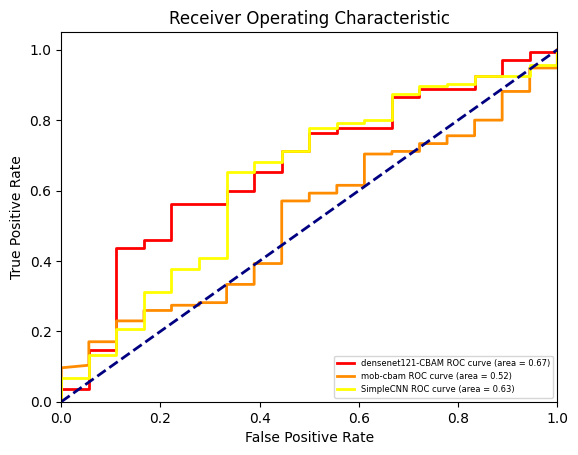

Supplement: Supplementary file 3 [file DataSheet3.zip › Sp1.densenet121-cbam、mob-cbam、cnn/binary-TN.png]

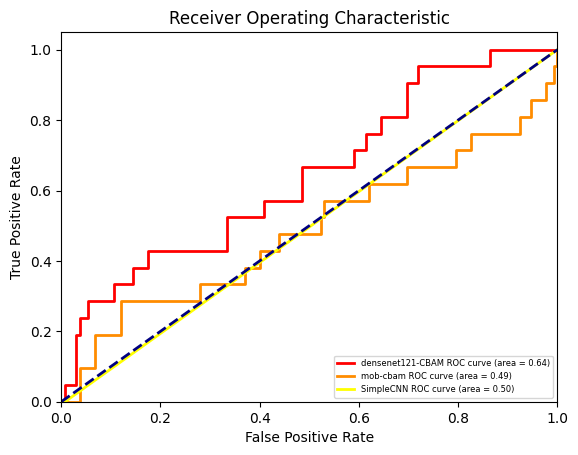

Supplement: Supplementary file 3 [file DataSheet3.zip › Sp1.densenet121-cbam、mob-cbam、cnn/multi-HER2HR+.png]

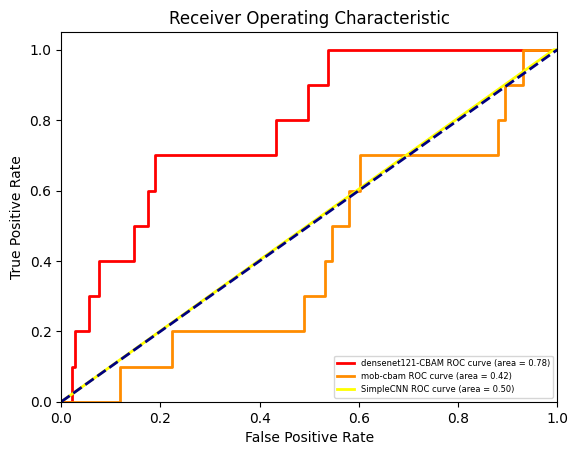

Supplement: Supplementary file 3 [file DataSheet3.zip › Sp1.densenet121-cbam、mob-cbam、cnn/multi-HER2HR-.png]

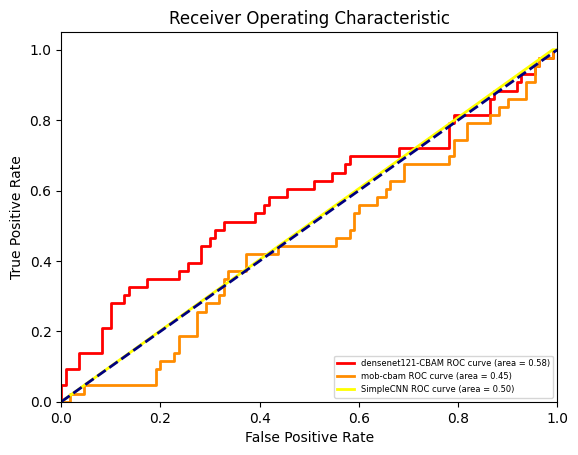

Supplement: Supplementary file 3 [file DataSheet3.zip › Sp1.densenet121-cbam、mob-cbam、cnn/multi-LuminalA.png]

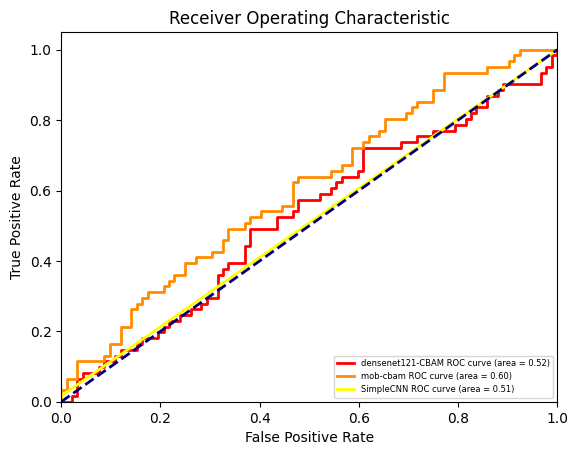

Supplement: Supplementary file 3 [file DataSheet3.zip › Sp1.densenet121-cbam、mob-cbam、cnn/multi-LuminalB.png]

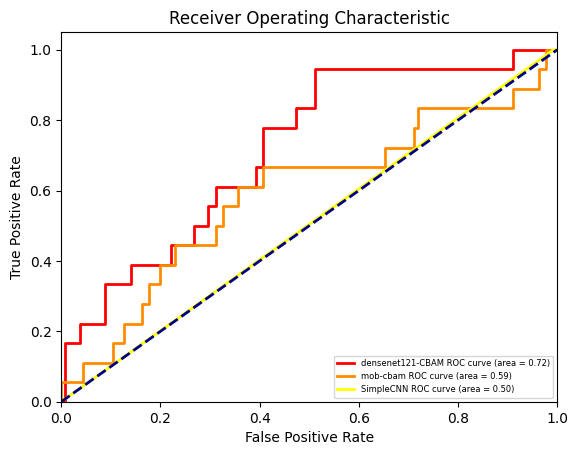

Supplement: Supplementary file 3 [file DataSheet3.zip › Sp1.densenet121-cbam、mob-cbam、cnn/multi-TN.png]

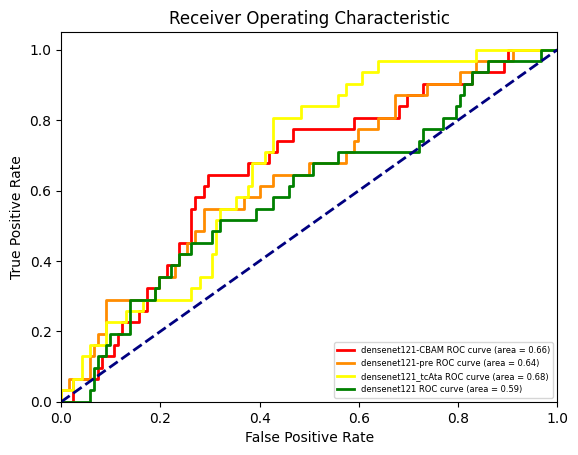

Supplement: Supplementary file 4 [file DataSheet4.zip › Sp2.densenet121-series/binary-HER2.png]

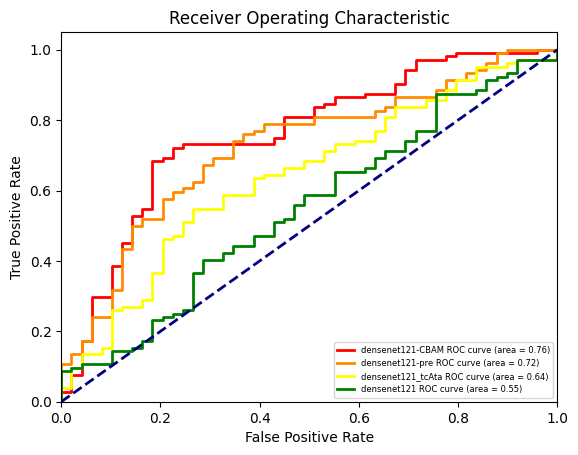

Supplement: Supplementary file 4 [file DataSheet4.zip › Sp2.densenet121-series/binary-Luminal.png]

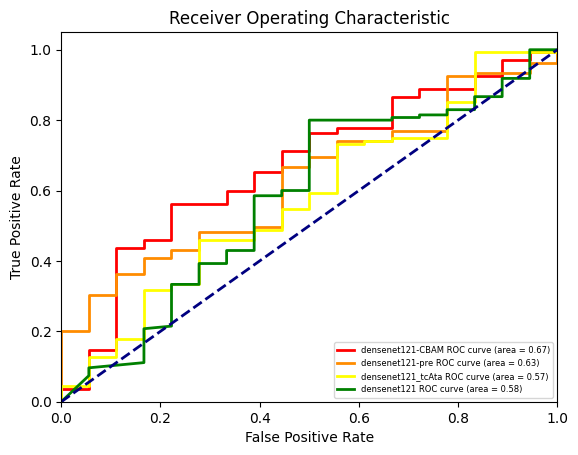

Supplement: Supplementary file 4 [file DataSheet4.zip › Sp2.densenet121-series/binary-TN.png]

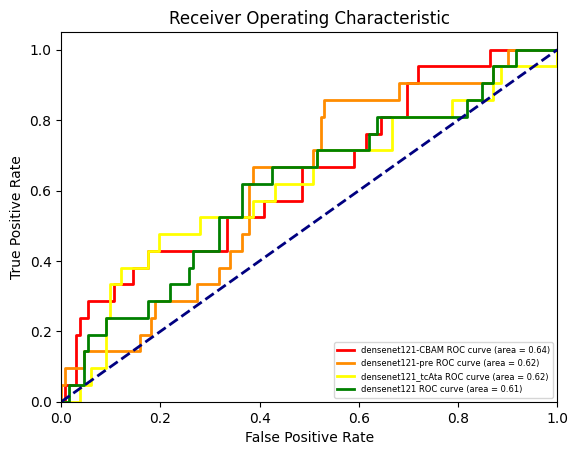

Supplement: Supplementary file 4 [file DataSheet4.zip › Sp2.densenet121-series/multi-HER2HR+.png]

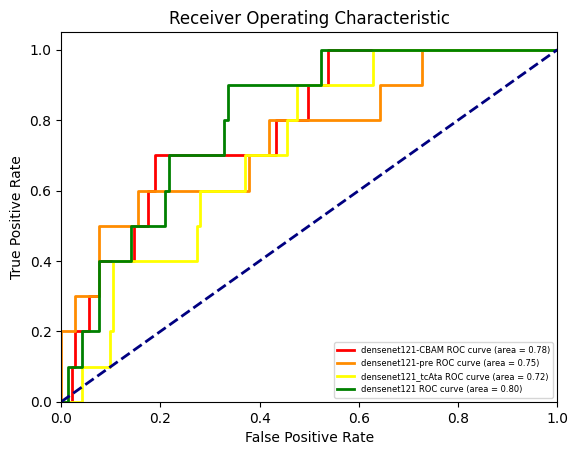

Supplement: Supplementary file 4 [file DataSheet4.zip › Sp2.densenet121-series/multi-HER2HR-.png]

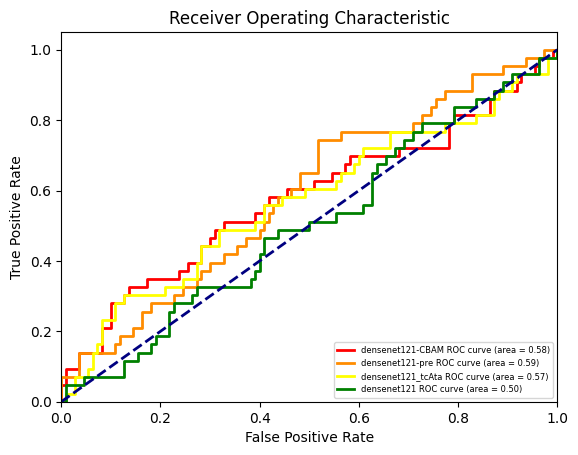

Supplement: Supplementary file 4 [file DataSheet4.zip › Sp2.densenet121-series/multi-LuminalA.png]

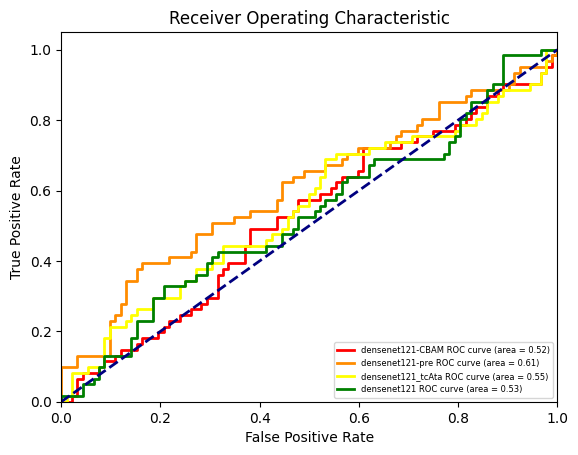

Supplement: Supplementary file 4 [file DataSheet4.zip › Sp2.densenet121-series/multi-LuminalB.png]

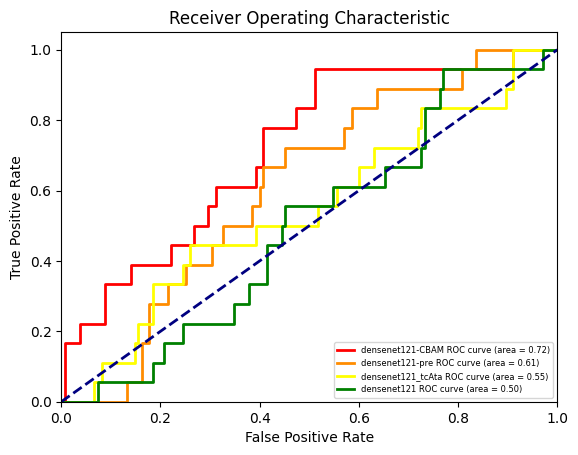

Supplement: Supplementary file 4 [file DataSheet4.zip › Sp2.densenet121-series/multi-TN.png]
