# Supplementary material for: Predicting molecular subtype in breast cancer using deep learning on mammography images
Source: Front Oncol. 2025 Sep 16;15:1638212. doi: 10.3389/fonc.2025.1638212 (PMC12479247; doi:10.3389/fonc.2025.1638212)
Supplement: Supplementary file 5 [file DataSheet5.pdf]

Supplementary Figure 5. Comparative visualization for each binary classification task

Visualization of progressive attention patterns across network layers (left to right, top to bottom): Original Image、Attention Map（features.conv0）、Attention Map（features.denseblock1.0）、Attention Map（features.denseblock1.1）、Attention Map（transition1）、Attention Map（features.denseblock2.0）、Attention Map（features.denseblock2.1）、Attention Map（transition2）、Attention Map（features.denseblock3.0）、Attention Map（features.denseblock3.1）、Attention Map（transition3）、Attention Map（features.denseblock4）

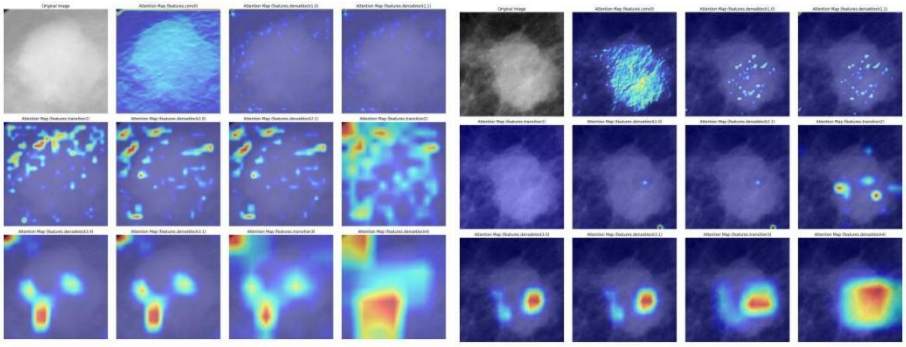

Supplementary Figure 5 (A). Attention maps generated by DenseNet121-CBAM for a non-TN subtype patient's mammographic CC (left) and MLO (right) views.

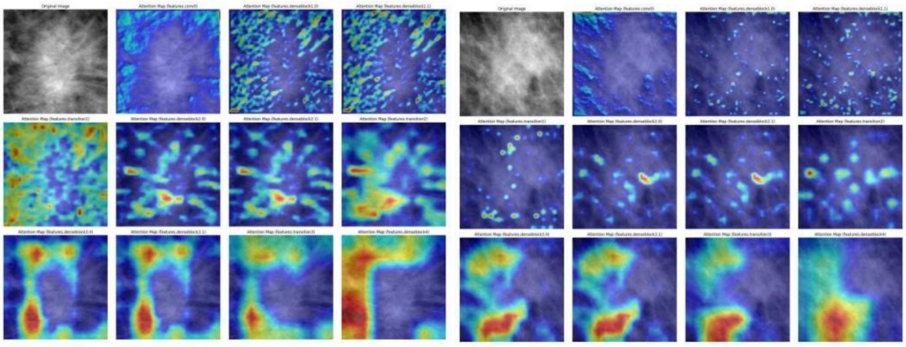

Supplementary Figure 5 (B). Attention maps generated by DenseNet121-CBAM for a TN subtype patient's mammographic CC (left) and MLO (right) views.

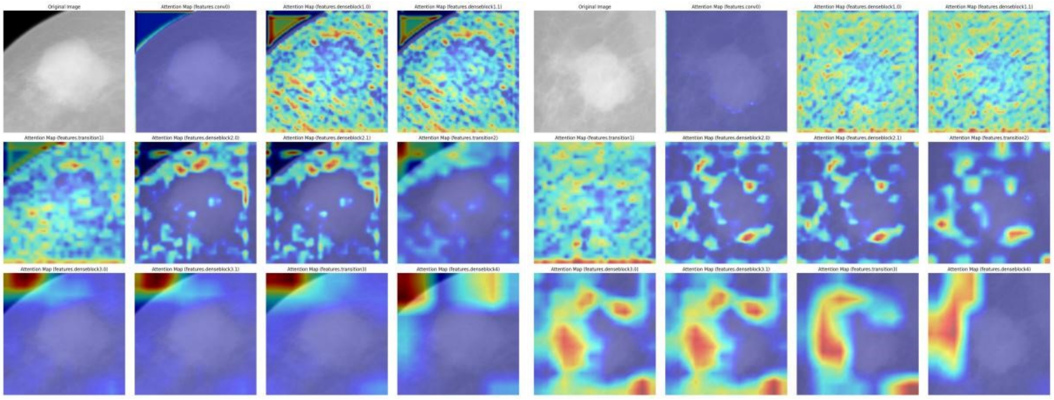

Supplementary Figure 5 (C). Attention maps generated by DenseNet121-CBAM for a non-Luminal subtype patient's mammographic CC (left) and MLO (right) views.

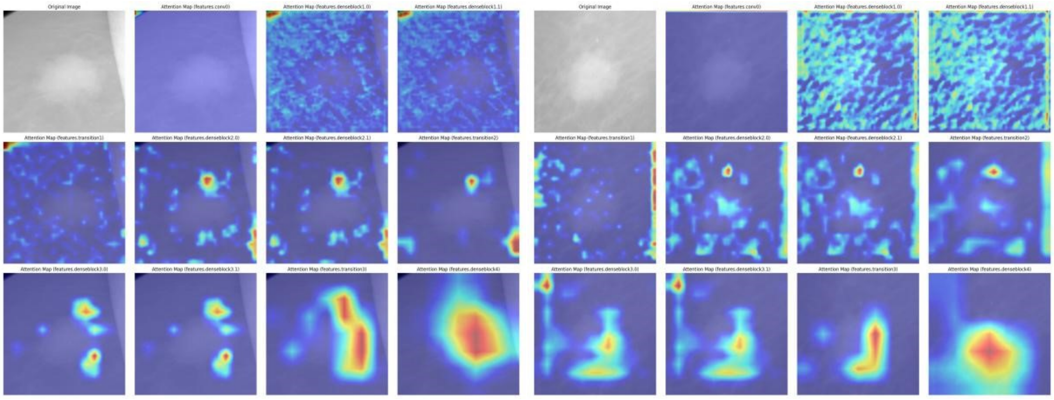

Supplementary Figure 5 (D). Attention maps generated by DenseNet121-CBAM for a Luminal subtype patient's mammographic CC (left) and MLO (right) views.

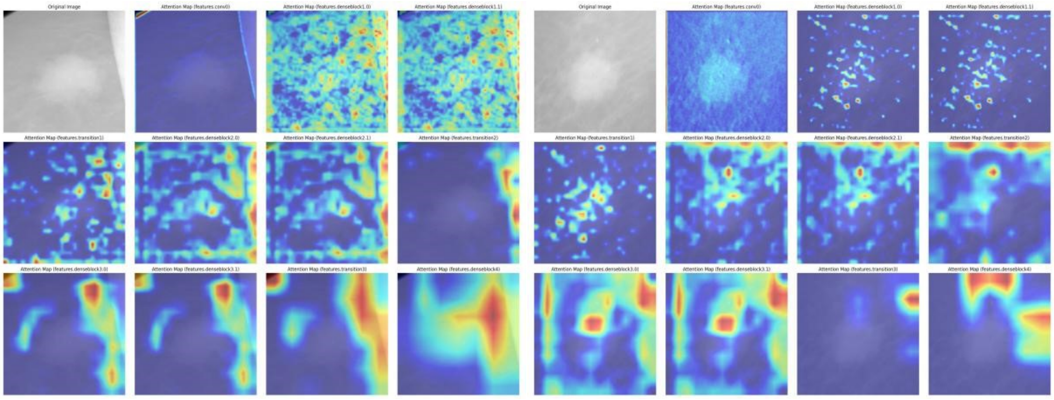

Supplementary Figure 5 (E). Attention maps generated by DenseNet121-CBAM for a non-HER2 subtype patient's mammographic CC (left) and MLO (right) views.

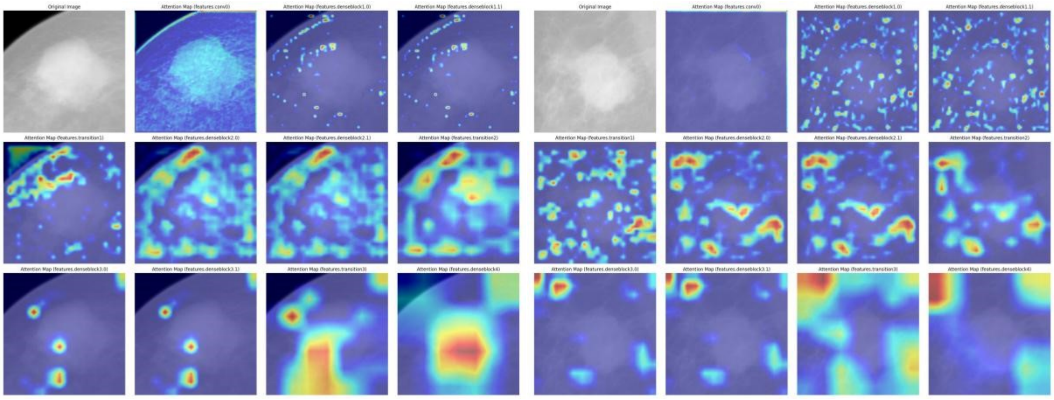

Supplementary Figure 5 (F). Attention maps generated by DenseNet121-CBAM for a HER2 subtype patient's mammographic CC (left) and MLO (right) views.
